# Current and Projected Future Spatial Distribution Patterns of *Prunus microcarpa* in the Kurdistan Region of Iraq

**DOI:** 10.3390/biology14040358

**Published:** 2025-03-30

**Authors:** Renas Y. Qadir, Nabaz R. Khwarahm

**Affiliations:** 1 Department of Natural Resources, College of Agricultural Engineering Sciences, University of Sulaimani, Sulaimani 334, Kurdistan Region, Iraq; 2 Department of Biology, College of Education, University of Sulaimani, Sulaimani 334, Kurdistan Region, Iraq

**Keywords:** climate change, Iraq, ecological distribution, MaxEnt, *Prunus microcarpa*

## Abstract

*Prunus microcarpa* is an endemic species prevalent throughout the highlands of the Kurdistan Region of Iraq. Conservation, introduction, and restoration efforts require an in-depth understanding of the species’ current and future habitat distributions under different climate change scenarios. This study utilized field observations, species distribution modeling, geospatial techniques, and environmental predictors to analyze the distribution and forecast potential habitats for *P. microcarpa* in the highlands of Iraq. Findings indicate that, according to the global climate models (i.e., BCC-CSM2-MR and MRI-ESM2.0), the reduction in habitat for the species is projected to be more than the potential expansion. Specifically, the area of habitat is expected to reduce by 2351.908 km^2^ (4.6%) and 2216.957 km^2^ (4.3%), while it could increase by 1306.384 km^2^ (2.5%) and 1015.612 km^2^ (2.0%) for the respective climate models. Topographic features such as elevation and slope, climatic conditions, precipitation seasonality, and annual mean temperature relatively shape the distribution of *P. microcarpa*. The modeling demonstrated good predictive capability (area under the curve (AUC) score = 0.933). The total study area is approximately 51,558.327 km^2^, with around 20.5% (10,602 km^2^) identified as suitable habitat for *P. microcarpa*. These findings offer essential baseline information for conservation strategies and provide new insights into where the species currently resides and where it could be found in the future. This underscores how combining distribution modeling with geospatial techniques can be effective, particularly in data-deficient regions like Iraq.

## Simple Summary

*Prunus microcarpa*, the small-fruited cherry, a unique plant found only in the highlands of Iraq’s Kurdistan Region, is at risk due to climate change. Protecting and restoring this species requires knowing where it currently grows and predicting how its habitat might shift in the future. This study aimed to map the plant’s current habitats and predict how climate change could influence its distribution. For this, we used field data, computer models, and environmental factors like elevation, rainfall patterns, and temperature to forecast future changes. Climate change is projected to shrink the plant’s habitat by 4.3–4.6% (about 2200–2350 km^2^), while new suitable areas might expand by only 2.0–2.5% (1015–1300 km^2^). Overall, the species is predicted to lose more habitat than it gains. These findings give conservationists and policymakers a clear plan to protect existing habitats, restore damaged areas, and prepare for climate impacts. Saving *P. microcarpa* helps preserve biodiversity, maintain healthy ecosystems, and support sustainable land use in Iraq’s highlands.

## 1. Introduction

The genus *Prunus* encompasses over 200 species of shrubs and trees, many of which thrive in temperate regions worldwide [1]. *Prunus microcarpa* is a highly branched shrub, typically reaching heights of 0.5 to 3.5 m. Its bark transitions from brown in younger stages to greyish or blackish tones as it matures, becoming smooth (glabrous) or nearly smooth (subglabrous) [2]. The plant flowers between March and May [3], producing small cherry-like fruits that ripen from May to July [2]. These fruits, locally termed Halaluk or Bilaluk [3], have a mildly bitter yet refreshing taste. Taxonomically, *P. microcarpa* belongs to a genus, a group of stone fruit species, within the Rosaceae (rose) family, many of which are nutritionally significant. The Rosaceae family also includes wild fruit-bearing genera such as hawthorn (*Crataegus monogyna* Jacq. and *C. azarolus* L.) and wild blackberries (*Rubus* spp.), whose edible fruits are common in local markets. *P. microcarpa* was first described by botanist C. A. Meyer in 1831 from specimens collected in the eastern Trans-Caucasus, specifically on Beschbarmak Mountain north of Baku [4]. Genetically, *P. microcarpa* is similar to cherries and demonstrates a superior ability to adapt to varying hot, cold, and dry climate conditions, as well as possessing resistance to salinity [5,6]. *P. microcarpa* species can thrive in rocky, arid soils, and are characterized by their small, densely hairy leaves, which demonstrate their ability to withstand drought [7]. This plant favors environments like the understory of oak and pine forests, hedgerows, and regions of coppiced or disturbed woodlands. It can thrive in diverse settings and soil types, flourishing in limestone, red marl, rocky terrains, deep moist soil, and dry slopes [2].

In the Kurdistan Region of Iraq (KRI), *P. microcarpa* grows at elevations between 600 and 2000 m. This species is prevalent in the forested regions of Iraq and can also be found in the mountainous areas of Iraqi Kurdistan, including sites like Jabal Sinjar, Jabal Bakhair, Mar Yaqub, near Simel, Zawita, Sarsang, the slopes of Gara Daqh, Harir, Gali Ali Beg, Marmarut, Haji Umran, Pira Magrun, Qopi Qaradagh, Jabal Avroman, above Darimar, and Penjwin [2]. The young branches of this plant are utilized for crafting canes, while the woody inner wall of the fruit is used to make prayer beads. Additionally, the fruit contains pectin, and the crushed ripe seeds serve as medicinal remedies for diarrhea, with small amounts also serving as a laxative in the local Ballakayati district of Iraq [8]. According to earlier research conducted in the KRI, the fruit of *P. microcarpa* is thought to contain the highest concentration of flavonoid glycoside (rutin) and fatty acids (lauric, myristic, palmitic, palmitoleic, stearic, oleic, linolenic, and erucic), which is advantageous for human health [9]. This species was chosen due to its cultural, medicinal, and ecological importance, as well as its ability to thrive in rocky, mountainous environments.

In recent decades, climate change has had significant impacts on plants and forest ecosystems, contributing to issues such as overuse, insect infestations, disease outbreaks, drought, and wildfires. Rainfall patterns and rising temperatures may significantly affect plant species distribution in certain regions [10]. Adverse climate change effects have been observed in Iraq, particularly through recurring drought events [11], fire incidents [12], land degradation [13], biodiversity loss [14], and disease outbreak [15]. Investigating how climate change influences species dispersal and mitigating its effects on forest ecosystems is crucial. In this context, assessing how climate change would impact the individual endemic plant species is imperative for evaluating the overall health of the forest ecosystem. A useful method for assessing the effects of climate change on plant populations is through species distribution models (SDMs). The aim of SDMs is to determine the potential areas within a region that can support the survival of a species. This prospective area is created by analyzing conditions from various ecologically suitable locations where the species thrives and persists [16]. SDMs predict the spatial distribution of species using either presence-only or presence/absence data from a defined location, along with the environmental characteristics of that site. The information, which encompasses locations and environmental factors, functions as the dependent and independent variables for forecasting species distribution [17]. One of the frequently used SDMs is maximum entropy (MaxEnt). The MaxEnt model is widely utilized for predicting species distribution because it provides several benefits, including the ability to work with categorical data and effectively predict species distribution even when data sets are incomplete or sample sizes are small [18]. MaxEnt relies solely on presence data and examines the environmental layer values at occurrence locations compared to those at background sites to create a habitat suitability map [19]. Ecological research is crucial for grasping the connection between environmental factors and the distribution of certain species. In light of climate change in the KRI, these studies can provide a basis for forecasting the future distribution patterns of *P. microcarpa*. This study employs the MaxEnt model to (1) assess the current distribution of *P. microcarpa* in the KRI through field research; (2) create a map of its known distribution within the province; and (3) forecast potential distribution changes while analyzing the effects of future climate change on *P. microcarpa*’s distribution and identifying the environmental factors that affect it. To accomplish this, we examined species presence data alongside different environmental variables—such as bioclimatic, soil, and topographic factors—and considered two climate change scenarios (SSP1-2.6 and SSP5-8.5) derived from two General Circulation Models (GCMs) for two future time frames: 2041–2060 and 2081–2100. The findings of this study may enhance our understanding of species distribution in response to climate change.

## 2. Materials and Methods

### 2.1. Study Area

**Figure 1:**
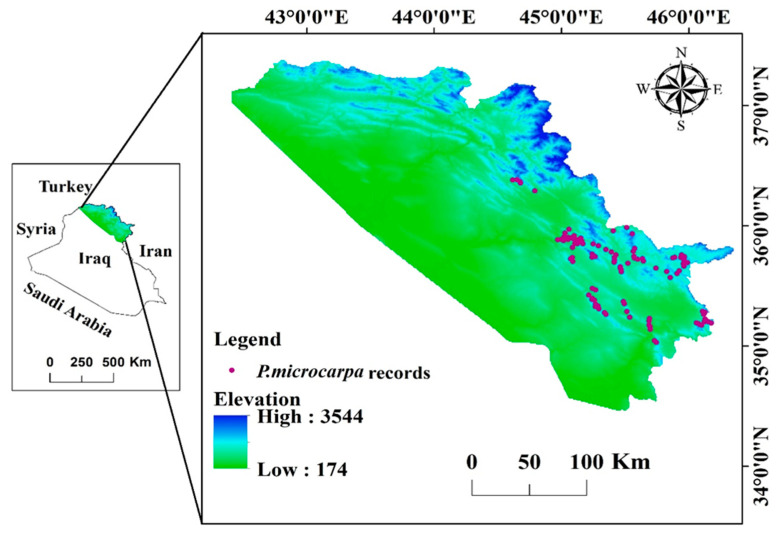
The modeled area, the Kurdistan Region of Iraq, with the occurrence point records of *P. microcarpa* in the Sulaimani Governorate (surveyed areas).

Iraq is one of the Middle Eastern countries and has an area of 438,320 km^2^. It has borders with Iran, Jordan, Turkey, and Saudi Arabia [20] (Figure 1). Iraq consists of four unique topographic regions determined by their land terrain: the mountainous area in the northeast (Kurdistan Region), the Western Plateau, the Jazera, and the Mesopotamian plain, as well as the Plateau and Hills Regions [2]. In Iraq, natural forests account for 4% of the country’s overall land only, predominantly found in the (KRI). This study uses the KRI as its defined boundary because the target species only occur in this region. The highlands are generally inaccessible and desolate, with altitudes ranging from ~800 to 3544 m above sea level [21]. Data on species occurrences were obtained in the Sulaimani Governorate (which is largely mountainous). The geographic coordinates for Sulaymani city are 35°33′40″ N latitude and 45°26′14″ E longitude. The province covers a total area of 17,023 square kilometers [13]. Sulaymani has a climate typical of its region, marked by hot, dry summers averaging 31.5 °C, while winters are notably colder, wetter, and windier, often experiencing occasional snowfall, with average temperatures around 7.6 °C. Annual rainfall usually varies between 400 and 600 mm.

### 2.2. Species Data

Surveys were conducted in the Sulaimani Governorate, spanning eight districts (Sharbazher, Penjwin, Mawat, Rania, Dukan, Darbanikhan, Qadaragh, and Halabja) in the KRI, from 17 August to 5 November 2024. The aim was to collect presence data for *P. microcarpa* using a hand-held GPS device (GARMIN-ETREX 22X). Furthermore, to cover the research region, random sampling stratified strategy was utilized [22]. The surveys performed for this research identified 127 species occurrence points for *P. microcarpa*. However, after implementing pre-processing steps like eliminating duplicates and applying spatial adjustments, this figure decreased to 105 points, which were kept for further analysis. Additionally, to minimize spatial autocorrelation among the sites, a spatial filter of at least 1 km was utilized on the dataset [20]. Applying the spatial filtering technique aids in reducing sampling errors and improves the variability in elevation differences among locations. Quality assurance was conducted using ArcGIS 10.8 in conjunction with the enhanced SDMtoolbox 2.5 [23].

### 2.3. Environmental Variables

Three categories of datasets concerning environmental features, including topography and climate, have been recognized as essential factors that significantly affect the distribution of tree species [24]. Natural populations of the species have solely been identified in the highlands of Iraq, with no other areas providing a habitat. As a result, we concentrated our research on the KRI for our distribution modeling. The existing records for *P. mirocarpa* were located within the highland boundaries of the KRI. Our initial dataset included a range of bioclimatic variables for both current and future climate scenarios, with the most recent climatic data obtained from the World Climate database [25] (www.worldclim.org, accessed on 13 November 2024); likewise, climate variables were obtained by analyzing the spatial distribution of presence records and the KRI boundary, which served as the potential range for the target species. This research employed 19 bioclimatic variables (Table 1) to model existing climatic conditions (from 1970 to the 2000s) and future climate scenarios for the years 2041–2060 and 2081–2100, using two shared socioeconomic pathways (SSP 126 and SSP 585) [26]. The modeling process utilized the BCC-CSM2-MR climate system model developed by the Beijing Climate Center of the China Meteorological Administration, along with version 2.0 of the Meteorological Research Institute Earth System Model (MRI-ESM2.0) [27]. Topographic data, including the digital elevation model (DEM), slope, and aspect, were sourced from the Shuttle Radar Topography Mission (SRTM) (http://srtm.csi.cgiar.org/srtmdata, accessed on 13 December 2024). In total, six bioclimatic variables (bio1, bio2, bio4, bio12, bio14, and bio15) listed in Table 1, alongside other environmental factors like the DEM and slope, were utilized to map the current and future distribution patterns of *P. microcarpa*. The spatial resolution of all environmental variables is 1 km.

### 2.4. Model Building

Throughout this effort, the maximum entropy method model (MaxEnt Version 3.4.3) [19] was employed to forecast the geographic distribution of *P. microcarpa* in the KRI. MaxEnt relies solely on presence data along with conditioning factors [28]. The algorithm calculates the likelihood of species occurrence, taking environmental constraints into account [29]. The model is acknowledged as one of the most effective methods [30]. We used 80% of the species data for training and 20% for testing, while keeping the remaining parameters at their default levels, as instructed by [31]. This leads to generally acceptable outcomes. Additionally, various factors can influence the reliability of the model’s outputs, including the modeler’s knowledge of the research region, the data points used, and the parameter settings of the model. Nonetheless, using the default settings does not guarantee precise models [32]. As a result, the study conducted 500 iterations and opted for 10 model replicates. These 10 replicates produced reasonable suitability maps (i.e., average maps). Additionally, the number of chosen background points was set at 5000 [33]; this was seen as sensible relative to the (n = 105) records of *P. microcarpa*. In the model development process, the default value of 1 for the (β = regularization multiplier) in MaxEnt was chosen [19,32]. To evaluate the relative significance and impact of the predictors on the likelihood of *P. microcarpa*’s habitat distribution, the jackknife test was chosen [34]. Based on the ‘maximum test sensitivity plus specificity’ [35], a threshold value of 0.2 was applied to differentiate between areas of suitable habitat (i.e., ≥0.2) and unsuitable habitat (i.e., <0.2) for the distribution of *P. microcarpa*, representing the likelihood of its occurrence [36]. The threshold value was established based on the average of 10 models. The classification involved dividing the continuous map into four categories, with the suitability ratings as follows: 0–0.2 (unsuitable), 0.2–0.41 (low suitability), 0.41–0.62 (moderate suitability), and 0.62–0.83 (high suitability).

### 2.5. Model Evaluation

The area under the curve (AUC) of the receiver operating characteristic (ROC) curve is a measure utilized to assess model performance by comparing correct and incorrect predictions at various thresholds [37]. The threshold value was established by averaging the results from 10 models (10 replicates). The classification process involved transforming the continuous map into four distinct categories [38]. The AUC value varies from 0 to 1, where higher values (ranging from 0.7 to 1.0) signify that the model effectively differentiates between outcomes, outperforming random guessing. In contrast, a lower AUC value suggests poorer model performance, with values under 0.5 reflecting weak capability [38].

### 2.6. Current and Future Change Analysis

The model’s forecasts for habitat distribution changes, both currently and in the future, were assessed using habitat suitability maps. The ArcGIS platform’s spatial tools were used to analyze and compare the distributions of current and future habitats. The species’ distribution changes were classified into four classes: (i) range expansion, suggesting areas likely to become suitable for *P. microcarpa* in the future; (ii) unsuitable, referring to areas that are currently unsuitable and will remain so; (iii) no change, including regions already occupied by *P. microcarpa* that will continue to be occupied; and (iv) range contraction, denoting areas where *P. microcarpa* is expected to decline in the future.

## 3. Results

### 3.1. Distributions and Variable Importance

**Figure 2:**
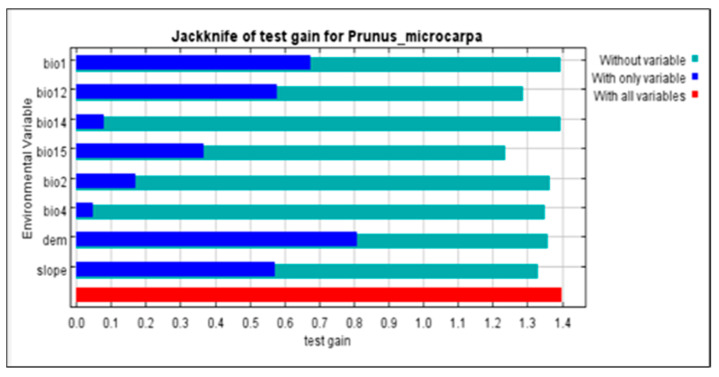
Environmental factors are evaluated using the jackknife test, regularized gains, and AUC gain for *P*. *microcarpa*.

The digital elevation model (DEM) accounted for 31.8% of the contribution, indicating its significant impact on the distribution of *P. microcarpa* and (bio15, precipitation seasonality), slope, and (bio12, annual precipitation) contributed 23%, 21.6%, and 10%, respectively, making these four factors responsible for nearly 86.4% of the overall contributions. In contrast, (bio14, precipitation of the driest month), (bio4, temperature seasonality), and (bio1, annual mean temperature) had the least impact on the distribution of *P. microcarpa* in the KRI, as shown in Table 2. The jackknife test, which helps standardize training gains, showed that DEM, (bio12), and (bio1) provided more valuable data for assessing the distribution arrangement of *P. microcarpa* compared to other predictors (Figure 2).

### 3.2. The Model’s Performance

**Figure 3:**
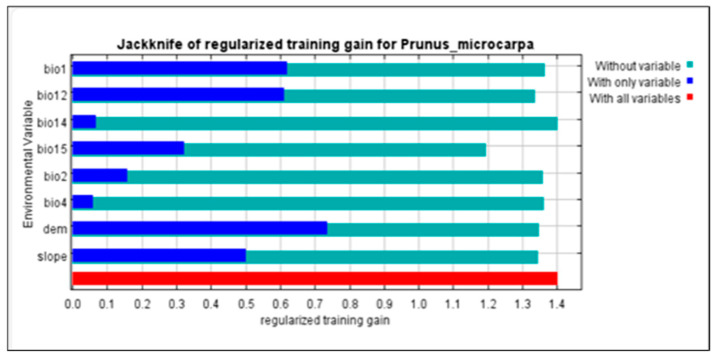
The model’s performance, average AUC, and the standard deviations for *P. microcarpa*.

In this study, the model was deemed excellent due to its AUC > 0.90, see (Figure 3), with the performance for *P. microcarpa* demonstrated by an AUC of 0.933 ± 0.006. This value, which is near one, along with AUC values exceeding 0.75, suggests a strong fit for the model employed [39]. The model demonstrated significant precision in delineating suitable versus unsuitable habitats.

### 3.3. Current and Future Change Analysis for P. microcarpa

**Figure 4:**
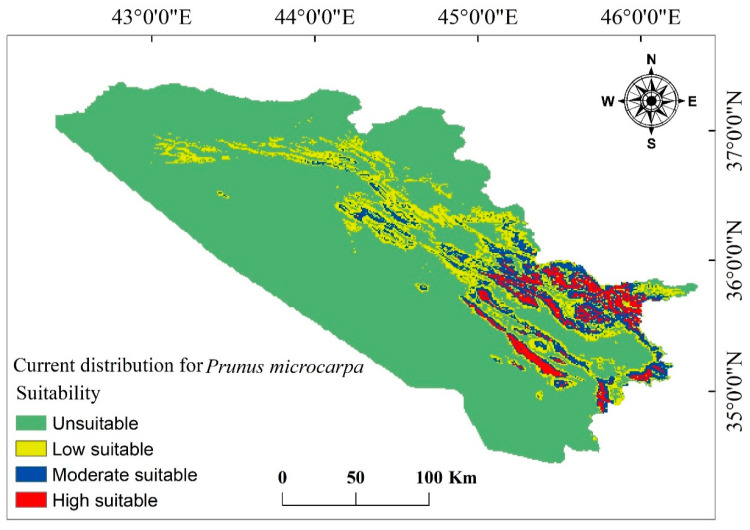
The habitat suitability for *P. microcarpa* was assessed by analyzing specific environmental factors and occurrence data.

**Figure 5:**
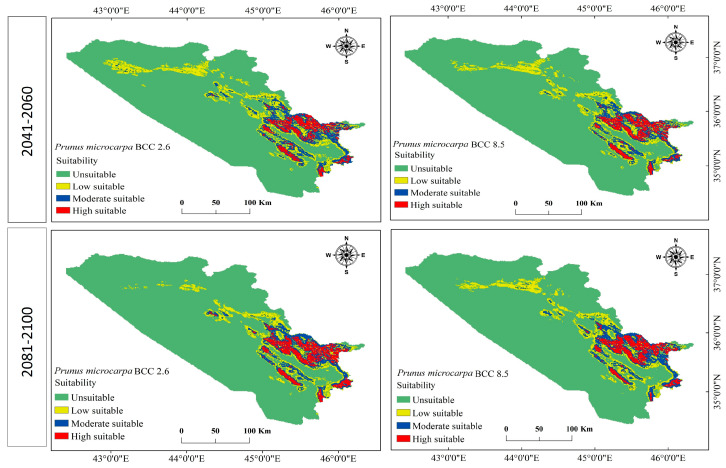
Future habitat distribution for *P. microcarpa* in the BCC-CSM2-MR model for the 2041–2060 and 2081–2100 periods, based on the 2.6 and 5.8 SSP scenarios.

**Figure 6:**
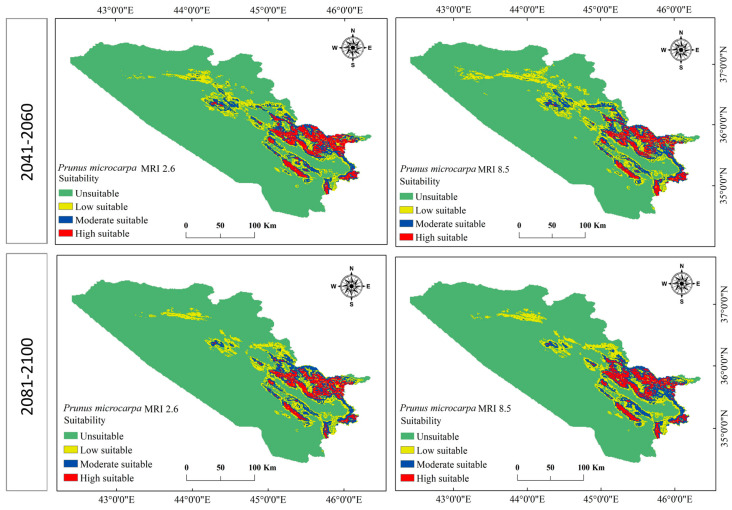
Future habitat distributions of *P. microcarpa* based on the MRI-ESM2.0 model under the 2.6 and 5.8 SSPs for the years 2041–2060 and 2081–2100.

The model effectively identified a suitable habitat distribution for *P. microcarpa* in the KRI based on the chosen environmental factors. It estimated that approximately 10,602.715 km^2^ (20.5%) of the entire study region of 51,558.327 km^2^ is appropriate for *P. microcarpa*, while around 40,955.612 km^2^ (79.4%) was deemed unsuitable (see Table 3 and Figure 4). Among the suitable habitat, the model categorized 5759.774 km^2^ (11.2%) as having low suitability, 3373.781 km^2^ (6.5%) as moderately suitable regions, and 1469.160 km^2^ (2.8%) as highly suitable areas. Future projections indicated that both unsuitable and highly suitable areas would increase under the BCC scenarios for the climate periods 2041–2060 and 2081–2100. Conversely, low and most moderate suitability habitats would decline (as shown in Table 3 and Figure 5). Under MRI scenarios, all categories remained consistent with BCC scenarios, except for a slight increase in the moderate habitat area during 2041–2060 for both SSPs (Table 4 and Figure 6). Overall, for both GCMs and under both SSP scenarios (2.6 and 2.8), the area classified as unsuitable is expected to expand between 2041 and 2100, while low and moderate suitability habitats will diminish. However, the habitat range for highly suitable areas would see a 1% expansion across all scenarios.

### 3.4. Current and Future Distribution Changes for P. microcarpa

**Figure 7:**
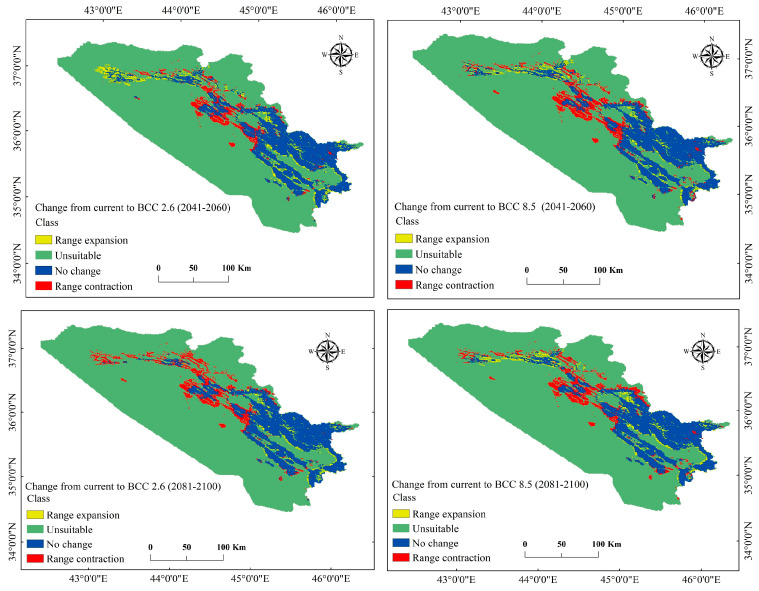
Comparison of the present distribution and future distribution according to the BCC-CSM2-MR model for the period of 2041–2100.

**Figure 8:**
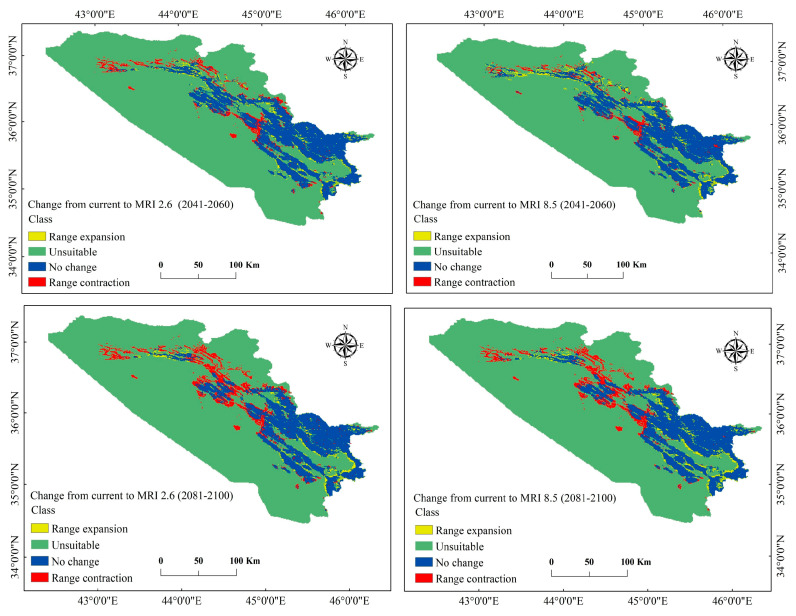
Difference between the present and future distribution using the MRI-ESM2.0 model for the period of 2041–2100.

The analysis of distributional changes over time and space for the scenarios (BCC 2041–2060 and 2081–2100) showed that *P. microcarpa* would experience both spatial expansion and contraction, with the latter being more significant. For instance, under BCC 2.6 and 8.5 for the period 2041–2060, the range of *P. microcarpa* contracted by 3.6% (1837.841 km^2^) and 4.7% (2443.035 km^2^), respectively. In the subsequent period of 2081–2100, the habitat would further contract by 5.0% (2585.638 km^2^) and 4.6% (2351.908 km^2^), respectively. In contrast, under BCC 2.6 and 8.5 during 2041–2060, there was an expansion of 3.2% (1674.369 km^2^) and 2.0% (1046.915 km^2^). In the later years of 2081–2100, the habitat would expand by 1.4% (706.755 km^2^) and 2.5% (1306.384 km^2^) (Table 5 and Figure 7). Modeling scenarios under MRI 2.6 and 8.5 for 2041–2060 similarly indicated habitat contractions of 1506.724 km^2^ (2.9%) and 1215.257 km^2^ (2.4%), with expansions of 991.961 km^2^ (1.9%) and 1350.208 km^2^ (2.6%). For the 2081–2100 period, expansions were 1.6% (800.664 km^2^) and 2.0% (1015.612 km^2^), while contractions reached 4.9% (2523.032 km^2^) and 4.3% (2216.957 km^2^) (Table 6 and Figure 8). Overall, the most significant habitat expansion for the species was observed at 3.2% (1674.369 km^2^) under the BCC 2.6 scenario for 2041–2060, while the largest contraction was noted for the BCC 2.6 scenario in 2081–2100 at 5.0% (2585.638 km^2^).

## 4. Discussion

### 4.1. P. microcarpa Distribution Pattern Under Current and Future Climate Conditions

A major factor leading to the fragmentation and degradation of ecosystems, along with a decline in biodiversity, is climate change [40]. To improve our comprehension of the effects of climate change on the distribution of plant communities in various ecosystems, SDMs are widely used. This study utilized MaxEnt modeling, which indicated that from 2041 to 2100, the category of unsuitability would increase under all shared socioeconomic pathways (SSPs) scenarios for the two global climate models (GCMs), BCC-CSM2-MR and MRI-ESM2.0, while low and moderately suitable habitats would diminish. Conversely, the highly suitable habitat range is expected to increase by 1% across all scenarios from 2041 to 2100. As a result, while species habitats may expand geographically, this growth will be less significant than the reduction in less suitable areas. Consequently, conservation efforts should prioritize regions where species face the greatest vulnerability, specifically in low and moderately suitable habitats. Analyzing the distribution of areas that may become climatically suitable in light of projecting future climate scenarios is crucial for developing effective long-term conservation plans for vulnerable and economically significant species [41]. Our findings support earlier research regarding the contraction of species ranges due to habitat loss, as well as their expansions, in the KRI [42] and other parts of the world [43,44,45,46]. For instance, in Iran, Naghipour et al. [47] reported that the suitable habitat for *Crataegus azarolus* L. is anticipated to shrink due to impending climate change. This research highlights that, despite ongoing degradation, *P. microcarpa* is still not widely distributed in the KRI. Nonetheless, significant threats from climate change and other human-related factors, including war, deforestation, and firewood collection in winter, persist. This study proposes the following recommendations: (i) to evaluate the adaptability of *P. microcarpa*, seeds should be introduced to high-elevation areas; (ii) reforestation initiatives should be implemented in mountainous regions, particularly in Sulaimani Province, to mitigate the risks of species decline; (iii) conservation efforts should focus on mountains, as these are vital habitats for the species; and (iv) it is essential to control human activities like deforestation by swiftly instituting a monitoring and management strategy. The research faced some limitations, particularly in certain mountainous regions of the KRI, especially in the Sulaimani Governorate, where sample collection was prohibited due to the area’s political situation. Additionally, the presence of landmines in other locations made sample collection unsafe.

### 4.2. Variables Controlling the Distribution of P. microcarpa

Environmental conditions influence the distribution of species in plant communities [22]. This study evaluated eight environmental parameters to forecast the regional distribution of *P. microcarpa* in the KRI. The analysis showed that the (DEM) was a significant environmental factor, contributing 31.8% to the likelihood of *P. microcarpa*’s distribution in the KRI. In addition, the important permutation variable was (bio15, precipitation seasonality). Similarly, (bio12, annual precipitation) and (bio1, annual mean temperature) influenced the distribution probability of *P. microcarpa*. High altitude places (mountain areas) are typically associated with substantial amounts of precipitation [48] and as a result, the temperature is lower than in the lowlands. The Zagros Mountains undergo significant variations in temperature and precipitation throughout the seasons, characterized by scorching, arid summers and cool, rainy winters. In the KRI region, the mountains can get up to 1200 mm of rainfall each year, most of which occurs between October and May [2]. As temperatures continue to rise in arid and semi-arid regions, the ability of plant species and ecosystems to adjust to changes in climate is expected to decrease [49]. Research indicates that appropriate habitats for key tree species will diminish and shift over time [50]. Optimal growing conditions for plant growth are anticipated to move to higher elevations where temperatures are cooler and rainfall is more abundant, fostering better environments for their development. Furthermore, studies have shown that plant species are migrating to higher altitudes, particularly in mountainous areas [51,52]. The distribution of plant species is influenced by factors such as climate tolerance, ecological needs, and relationships with other organisms. Climate change significantly endangers biodiversity by diminishing the habitats accessible to various species [53], ultimately altering their global distribution as plants depend on specific temperature ranges and sufficient moisture to thrive.

### 4.3. Uncertainties of the Model

Ecological predictions from MaxEnt modeling carry inherent constraints tied to factors like input data quality (e.g., climate projections and species occurrence records) and inconsistencies in the timing of datasets such as DEM and slope. Such mismatches can skew outcomes, for instance, by miscalculating how climate change alters habitat suitability. These challenges are often mitigated by refining species datasets, validating spatial boundaries, and optimizing model parameters. However, uncertainties may persist in data-scarce contexts, even after adjustments. Though MaxEnt performs reliably with limited data, its accuracy depends on how closely species observations align with the study area’s ecological context. In this work, these limitations were addressed by prioritizing geographic precision and employing regionally representative datasets.

## 5. Conclusions

The research focused on assessing the current and future distribution of *P. microcarpa* within the KRI. the findings are essential for shaping forest management strategies in Iraq’s highlands. The research explored how climate change impacts its geographic distribution by utilizing two scenarios from global climate models. Through the application of a machine learning algorithm and geospatial methods, the analysis generated categorical maps highlighting areas of current and potential habitat suitability. These insights are valuable for enhancing conservation efforts in both the study region and similar climatic zones. Moreover, forecasts suggest that in both climate change scenarios, the suitability of habitats for the species is likely to decrease considerably from 2041 to 2100, with habitat loss outweighing any possible benefits. These findings offer a roadmap for conservationists to protect existing habitats, restore degraded areas, and plan for climate impacts. By safeguarding *P. microcarpa*, communities can preserve biodiversity, support healthy ecosystems, and promote sustainable land use.

## Figures and Tables

**Table 1:** The environmental parameters taken into account throughout the modeling process. Only the variables highlighted in bold were used in this study. All of the precipitation and temperature related variable have (mm) and (°C) units.

Environmental Variable (Abbreviation)	Environmental Variable (Abbreviation)
**Annual Mean Temperature (bio 1)**	**Precipitation of Driest Month (bio 14)**
**Mean Diurnal Range (bio 2)**	**Precipitation of Seasonality (bio 15)**
Isothermality (bio 3)	Precipitation of Wettest Quarter (bio 16)
**Temperature Seasonality (bio 4)**	Precipitation of Driest Quarter (bio 17)
Max Temperature of Warmest Month (bio 5)	Precipitation of Warmest Quarter (bio 18)
Min Temperature of Coldest Month (bio 6)	Precipitation of Coldest Quarter (bio 19)
Temperature Annual Range (bio 7)	**Digital Elevation Model (m) DEM**
Mean Temperature of Wettest Quarter (bio 8)	**Slope (degree)**
Mean Temperature of Driest Quarter (bio 9)	Aspect (degree)
Mean Temperature of Warmest Quarter (bio 10)	
Mean Temperature of Coldest Quarter (bio11)	
**Annual Precipitation (bio12)**	
Precipitation of Wettest Month (bio13)	

**Table 2:** The contribution percentage and “permutation importance” of the predictors utilized in the modeling process.

Variable	Contribution %	Permutation Importance %
dem	31.8	10.3
bio15	23	41.6
slope	21.6	5.6
bio12	10	19.7
bio2	4.9	4.3
bio14	4.6	0
bio4	3.2	4.4
bio1	0.8	13.9

**Table 3:** Mapped areas of habitat suitability and unsuitability for *P. microcarpa* in the BCC-CSM2-MR model for the present and future (as a percentage) under SSPs 2.6 and 8.5 for the periods 2041–2060 and 2081–2100.

Category	Current Distribution		Future Distribution							
			BCC CSM2 MR							
			2041–2060				2081–2100			
			SSP1 2.6		SSP5 8.5		SSP1 2.6		SSP5 8.5	
	Area (km^2^)	%	Area (km^2^)	%	Area (km^2^)	%	Area (km^2^)	%	Area (km^2^)	%
Unsuitable	40,955.612	79.4	41,119.084	79.8	42,351.731	82.1	42,834.495	83.1	42,001.136	81.5
Low suitability	5759.774	11.2	5055.802	9.8	4385.219	8.5	3507.341	6.8	4360.177	8.5
Moderate suitability	3373.781	6.5	3540.731	6.9	2877.105	5.6	2939.711	5.7	3195.005	6.2
High suitability	1469.160	2.8	1842.710	3.6	1944.272	3.8	2276.780	4.4	2002.008	3.9
Total	51,558.327	100	51,558.327	100	51,558.327	100	51,558.327	100	51,558.327	100

**Table 4:** Mapped areas of habitat suitability and unsuitability for *P. microcarpa* in the MRI-ESM2.0 model for the present and future (as a percentage) under SSPs 2.6 and 8.5 for the periods 2041–2060 and 2081–2100.

Category	Current Distribution		Future Distribution							
			MRI-ESM2.0							
			2041–2060				2081–2100			
			SSP1 2.6		SSP5 8.5		SSP1 2.6		SSP5 8.5	
	Area (km^2^)	%	Area (km^2^)	%	Area (km^2^)	%	Area (km^2^)	%	Area (km^2^)	%
Unsuitable	40,955.612	79.4	41,470.375	80.4	40,820.661	79.2	42,677.979	82.8	42,156.956	81.8
Low suitability	5759.774	11.2	4361.568	8.5	5345.877	10.4	4114.621	8.0	4196.009	8.1
Moderate suitability	3373.781	6.5	3416.910	6.6	3475.342	6.7	2987.709	5.8	3222.830	6.3
High suitability	1469.160	2.8	2309.475	4.5	1916.447	3.7	1778.017	3.4	1982.531	3.8
Total	51,558.327	100	51,558.327	100	51,558.327	100	51,558.327	100	51,558.327	100

**Table 5:** Comparison of the current and future distribution of *P. microcarpa* based on the BCC-CSM2-MR model for the period of 2041–2100.

Change Status	BCC CSM2 MR							
	2041–2060				2081–2100			
	Current-SSP1 2.6		Current-SSP5 8.5		Current-SSP1 2.6		Current-SSP5 8.5	
	Area (km^2^)	%	Area (km^2^)	%	Area (km^2^)	%	Area (km^2^)	%
Range expansion	1674.369	3.2	1046.915	2.0	706.755	1.4	1306.384	2.5
Unsuitable	39,281.243	76.2	39,908.697	77.4	40,248.857	78.1	39,649.229	76.9
No change	8764.874	17.0	8159.680	15.8	8017.077	15.5	8250.807	16.0
Range contraction	1837.841	3.6	2443.035	4.7	2585.638	5.0	2351.908	4.6
Total	51,558.327	100	51,558.327	100	51,558.327	100	51,558.327	100

**Table 6:** Comparison of the current and future distribution of *P. microcarpa* based on the MRI-ESM2.0 model for the period of (2041–2100).

Change Status	MRI-ESM2.0							
	2041–2060				2081–2100			
	Current-SSP1 2.6		Current-SSP5 8.5		Current-SSP1 2.6		Current-SSP5 8.5	
	Area (km^2^)	%	Area (km^2^)	%	Area (km^2^)	%	Area (km^2^)	%
Range expansion	991.961	1.9	1350.208	2.6	800.664	1.6	1015.612	2.0
Unsuitable	39,963.651	77.5	39,605.404	76.8	40,154.948	77.9	39,940.000	77.5
No change	9095.991	17.6	9387.458	18.2	8079.683	15.7	8385.758	16.3
Range contraction	1506.724	2.9	1215.257	2.4	2523.032	4.9	2216.957	4.3
Total	51,558.327	100	51,558.327	100	51,558.327	100	51,558.327	100

## Data Availability

The original contributions presented in this study are included in the article. Further inquiries can be directed to the corresponding author.
